# Loading of Porous Functionalized Calcium Carbonate Microparticles: Distribution Analysis with Focused Ion Beam Electron Microscopy and Mercury Porosimetry

**DOI:** 10.3390/pharmaceutics11010032

**Published:** 2019-01-15

**Authors:** Maryam Farzan, Roger Roth, Gabriela Québatte, Joachim Schoelkopf, Jörg Huwyler, Maxim Puchkov

**Affiliations:** 1 Division of Pharmaceutical Technology, Department of Pharmaceutical Sciences, University of Basel, Klingelbergstrasse 50, 4056 Basel, Switzerland; maryam.farzan@unibas.ch (M.F.); roger.roth@unibas.ch (R.R.); gabriela.quebatte@unibas.ch (G.Q.); joerg.huwyler@unibas.ch (J.H.); 2 Fundamental research, Omya International AG, 4665 Oftringen, Switzerland; joachim.schoelkopf@omya.com

**Keywords:** porous drug carrier, functionalized calcium carbonate, drug loading, focused ion beam scanning electron microscopy, mercury intrusion porosimetry, dipalmitoylphosphatidylcholine, bovine serum albumin

## Abstract

Accurate analysis of intraparticle distribution of substances within porous drug carriers is important to optimize loading and subsequent processing. Mercury intrusion porosimetry, a common technique used for characterization of porous materials, assumes cylindrical pore geometry, which may lead to misinterpretation. Therefore, imaging techniques such as focused ion beam scanning electron microscopy (FIB-SEM) help to better interpret these results. The purpose of this study was to investigate the differences between mercury intrusion and scanning electron microscopy and to identify the limitations of each method. Porous microparticles, functionalized calcium carbonate, were loaded with bovine serum albumin and dipalmitoylphosphatidylcholine (DPPC) by solvent evaporation and results of the pore size distribution obtained by both methods were compared. The internal structure of the novel pharmaceutical excipient, functionalized calcium carbonate, was revealed for the first time. Our results demonstrated that image analysis provides a closer representation of the material distribution since it was possible to discriminate between blocked and filled pores. The physical nature of the loaded substances is critical for the deposition within the pores of functionalized calcium carbonate. We conclude, that a combination of mercury intrusion porosimetry and focused ion beam scanning electron microscopy allows for a reliable analysis of sub-micron porous structures of particulate drug carriers.

## 1. Introduction

Porous microparticles are promising carriers for the delivery of a wide variety of substances. In particular, loading drugs into the porous structure of microparticles may increase the dissolution rate and the solubility of poorly soluble active pharmaceutical ingredients (API). This is achieved on one hand by enlarging the surface area, which is exposed to a dissolution medium, and on the other hand by increasing the internal energy of an API due to amorphization, because initial crystallization is inhibited by the restricted space inside the pore [1]. Microparticles can be used to deliver drugs to the site of action, for either systemic uptake or local treatment. Close contact between the microparticle and the site of action can be achieved by mucoadhesion and additionally, drug release can be modulated [2,3,4]. In contrast to nanoparticles, microparticles can be used for the production of solid dosage forms [5,6]. Orally administered microparticles are not absorbed into systemic circulation due to their size. Enteral administration of chemically inert and biodegradable drug carriers (such as, for example, calcium carbonate) is therefore considered to be safe [7,8].

A variety of different inorganic carriers have been explored for use in drug delivery. One of the most extensively used material in this field is calcium phosphate [9]. Other materials include titanium dioxide [10], alumina silicate [11], calcium carbonate [12], and silicon dioxide [13]. Porous calcium phosphate carriers in the form of scaffolds have been explored for local delivery of drugs and biologics to different tissues, e.g., bone tissue [14,15]. Porosity of such carriers directly influences the maximal achievable drug load (*DL*) [14,16].

Methods to load porous carriers with drugs include adsorption (e.g., fluidized bed processing), soaking, and solvent evaporation methods [2,17,18]. However, these methods often lead to deposition of substances on the surface of the carrier. This may lead to subsequent challenges in the manufacturing process due to modified surface properties. This includes altered flowability, compactability, and changes in the properties of the final product like delayed or retarded disintegration [19]. It would therefore be beneficial to load internal porous structures instead of surface deposition of drugs. This approach has additional advantages. Pore loading leads to taste masking and enhanced protection of sensitive substances against mechanical and oxidative stress. For example, shear stress was reported to induce loss of activity in enzymes and other protein-based drugs [20,21]. After loading, microparticles can be coated to provide an additional protection for sensitive cargo against enzymatic or microbial degradation and the harsh conditions within the gastrointestinal tract.

Drugs can be integrated into a particle during its synthesis, which has the advantage of a single step operation, but also brings a lot of variability into the final structure [22]. However, a more convenient strategy is to load substances into prefabricated inert carrier particles, which can be manufactured under controlled conditions and later can be combined with a variety of substances. However, due to the limited drug loading capacity of these structures, complete pore filling is important. In any case, sensitive analytical methods are needed to optimize loading protocols and to exploit the full capacity of porous structures. One of the problems for quantification of drug load is to distinguish between the fraction of intraparticle and extraparticle drug deposition. There are combinations of methods suggested, including thermal analysis, atomic force microscopy (AFM), sorption methods (i.e., BET), helium pycnometry, flow imaging microscopy, µCT etc. The BET sorption method is commonly used for characterization of porous structure and drug load. However, if the drug deposition on the surface leads to a blockage of pores, sorption methods will give an overestimation of drug load. In addition, if the deposited drug forms fractal surfaces on pore walls, overstated high specific surface areas will be registered [1]. Helium pycnometry could give more realistic results but has its own drawbacks such as helium entrapment inside pores [23]. Differential scanning calorimetry (DSC) has been used with the assumption that the drug in the pores does not melt or melts at lower temperature than the fraction of drug on the surface, due to smaller crystal size [24].

During the last few years, our group has investigated the properties of so-called functionalized calcium carbonate (FCC), which was recently introduced as a pharmaceutical excipient. It is a co-precipitate from calcium carbonate and calcium phosphate which are, depending on the type, mixed at different ratios ranging from 13 to 85% (*w*/*w*) calcium phosphate content and a porosity of approximately 60% (*v*/*v*) [25]. Exploring this new excipient led to several innovative drug delivery devices such as orally disintegrating or floating tablets [26,27,28], mucoadhesive delivery systems for colon targeting [2], delivery of proteins, and loading of various small molecule drugs [17,18]. For the mentioned applications [2,17,18,26,27,28,29], we have identified a major problem with drug loading into deep porous regions of investigated microparticles as well as the understanding of the internal structure. Markl et al. used a combination of terahertz pulse imaging, µCT, MIP and pycnometry together with an experimental setting for water sorption of FCC compacts. They interpreted the results as anisotropic pores that are perpendicular to the compaction direction of a tablet [30,31]. However, so far, no convincing data has been available which reveals the internal structure of single FCC particles.

**Figure 1:**
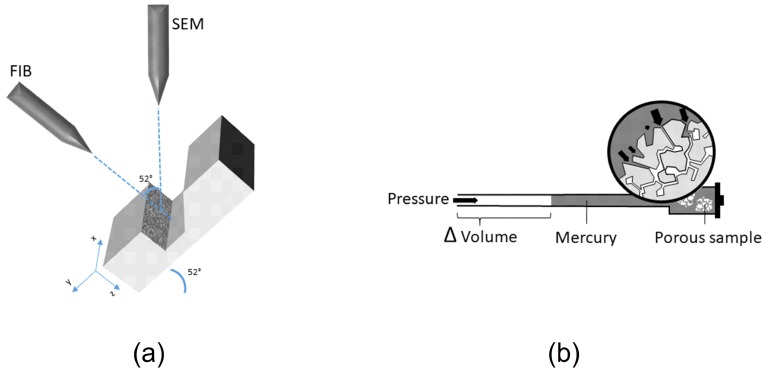
A schematic representation of focused ion beam scanning electron microscopy (FIB-SEM) and mercury intrusion porosimetry (MIP). (**a**) For FIB-SEM, a sample is tilted to be perpendicular to the ion (gallium) beam. The ion beam then mills a trench in the z direction of the sample, revealing a cross section, which is then imaged by the electron beam. (**b**) In an MIP setting, the pressure applied to the penetrometer pushes a non-wetting liquid (mercury) inside the pores of the sample. Pore size is calculated based on the pressure needed to force mercury into pores.

Therefore, this particular research aims to propose a method for most effective characterization of drug loading efficiency into porous materials composed of coprecipitated inorganic salts of calcium such as hydroxyapatite and calcium carbonate. The present study combines two analytical methods (i.e., mercury intrusion porosimetry (MIP) and focused ion beam scanning electron microscopy (FIB-SEM) to study the material distribution within the porous structure of FCC. Bovine serum albumin was used as a hydrophilic, high molecular weight compound. Dipalmitoylphosphatidylcholine (DPPC) was its small molecular weight and lipophilic counterpart. Compounds were loaded by solvent evaporation [17]. Although MIP (Figure 1b) is the most common technique for porosity analysis, there are some limitations, which should be considered when interpreting its results [32]. First, assuming a cylindrical pore shape leads to a wrong interpretation, since this technique measures only the largest pore opening (throat) and not the actual inner diameter of a cavity (body). Therefore, it is not possible to discriminate whether a volume attributed to a certain diameter corresponds to several small pores or one large pore with the same throat diameter. Second, MIP cannot measure inaccessible pores: a particle with blocked periphery pores shows the same internal volume and pore size distribution as a completely filled particle. Thus, MIP measures pore size distribution and skeleton density, but is not capable to distinguish between filled and blocked pores. MIP was therefore combined with FIB-SEM (Figure 1a) to visualize the true inner structure of FCC particles. In our experimental setup, a focused gallium ion beam was used to dissect FCC particles. The exposed inner surfaces were then visualized by SEM. It was thus possible to distinguish between blocked and filled porous structures. The results from FIB-SEM and MIP were combined to discuss the limitations and necessities of each method with respect to their capability to characterize drug loaded porous materials. Finally, previously unexplored porous structure of FCC was revealed and physical properties of the loading solutions, which affect the penetration into the porous particle were identified.

## 2. Materials and Methods

Omyapharm FCC (OG-500) was supplied by Omya International AG (Oftringen, Switzerland). Dipalmitoylphosphatidylcholine (DPPC) that has a phase transition temperature of 42 °C was kindly provided by Lipoid AG (Ludwigshafen, Germany). Bovine serum albumin (BSA) is commonly used as a standard reference protein in lab experiments. Heat shock fractionated BSA and Ph. Eur. grade methanol were purchased from Sigma Aldrich (St. Louis, MA, USA). Ultrapure water (<18.2 MΩ·cm resistivity) was obtained using a Milli-Q filtration station (Millipore, Billerica, MA, USA), calcium carbonate for density measurement was obtained from Lehmann and Voss and Co. (Hamburg, Germany). All substances were used as received without any further purification.

To calculate the intraparticulate porosity *P_I_* of the FCC, the particles were consolidated into a round, flat tablet with a diameter of 13 mm applying a pressure of 37 MPa. At this pressure, the interparticulate voids were not present anymore. The density of the tablet ρ*_T_* (kg/m^3^) was calculated using Equation (1):

(1) $$\rho_{T}=\frac{m_{T}}{V_{T}},$$

where *m_T_* is the mass and *V_T_* is the volume of the tablet. *P_I_* can then be calculated using Equation (2):

(2) $$P_{I}=1-\frac{\rho_{T}}{\rho_{S}},$$

where ρ*_S_* is the averaged (assuming 51:49 mass ratio [25]) skeletal density (2.95 kg/m^3^) of the two components hydroxyapatite and calcium carbonate, which are 3.16 [33], and 2.73 kg/m^3^, respectively [measured on a helium pycnometer (Accupyc 1330, Micromeritics, Norcross, GA, USA)].

The total intraparticulate pore volume *V_P_* (cm^3^) can be calculated using Equation (3):

(3) $$V_{P}=V_{T}-V_{S},$$

where *V_S_* is the volume of the skeleton structure. The volume of the skeleton structure is defined by Equation (4):

(4) $$V_{S}=\frac{m_{T}}{\rho_{S}},$$

### 2.1. Loading

FCC particles were loaded with DPPC and BSA at different drug loads (*DL*). The *DL* is described as the ratio between the loaded material and the total mass of the sample and was calculated by Equation (5):

(5) $$DL=\frac{m_{M}}{m_{M}+m_{FCC}},$$

where *m_M_* is the mass of the loaded material (g) and *m_FCC_* is the mass of FCC (g).

Low, medium and high loading formulations corresponding to 5% and 6.25%, 20% and 25%, and 30% and 37.5% (*w*/*w*) theoretical *DL*s for BSA and DPPC, respectively, were prepared by a solvent evaporation method as follows: Required amounts (Equation (5)) of DPPC and BSA were dissolved in 20 mL of methanol and ultrapure water, respectively. Afterwards defined amounts of FCC was added and dispersed in a 250 mL round bottom flask to achieve the theoretical *DL* (Equation (5)). Solvents were evaporated in a rotary evaporator (Rotavap R-114, Büchi, Flawil, Switzerland) at 60 rpm. The temperature of the preheated water bath was 50 °C (product temperature was 39 °C) and 40 °C (product temperature was 36 °C) and initial pressure was 300 mbar and 100 mbar for DPPC and BSA, respectively. Pressures were gradually reduced to 20 mbar and held at this pressure for 1 h and 2 h for DPPC and BSA, respectively to completely remove residual solvents. The dry product was gently ground in a mortar and sieved through a 355 μm sieve. All formulations were produced in triplicate.

### 2.2. Content Measurement by Thermogravimetry

The content of BSA and DPPC in FCC was measured with a thermogravimetric method. Samples were heated from 35 to 950 °C in a thermogravimetric analyzer (TGA7SDTA 851e, Mettler Toledo, OH, USA). The rate of heating was 10 °C/min. Pure DPPC, BSA, and unloaded FCC were used as references. Mass loss curves of separate components were recorded against temperature. Contents were calculated using the mass losses between 150 and 600 °C and the results were corrected for FCC mass loss in this temperature range.

The drug loads were calculated by Equation (6):

(6) $$DL=\frac{m_{M}}{m_{Tot}},$$

where *DL* is drug load (%), *m_M_* is mass of loaded material in the sample (mg) and *m_Tot_* is total initial mass of the sample (mg).

*m_M_* was calculated from the system of Equation (7):

(7) $$\{\begin{array}{c} m_{M}=m_{Tot}-m_{FCC}\\ \Delta m_{Tot}=m_{M}\times f_{M}+m_{FCC}\times f_{FCC} \end{array},$$

which yields:

(8) $$m_{FCC}=\frac{\Delta m_{Tot}-f_{M}\times m_{Tot}}{f_{FCC}-f_{M}},$$

where ∆*m_Tot_* is the total mass loss during the measurement (mg), *f_ref_* is the fraction of mass loss (%) that was measured for both, the loaded materials (pure BSA or DPPC) and FCC, respectively. *f_ref_* is defined in Equation (9):

(9) $$f_{ref}=\frac{\Delta m_{ref}}{m_{ref}},$$

where ∆*m_ref_* is mass loss during TGA (thermogravimetric analysis) in a temperature range of 150–600 °C, and *m_ref_* is the initial mass of the reference material (pure DPPC, BSA, or FCC).

### 2.3. Mercury Porosimetry

**Figure 2:**
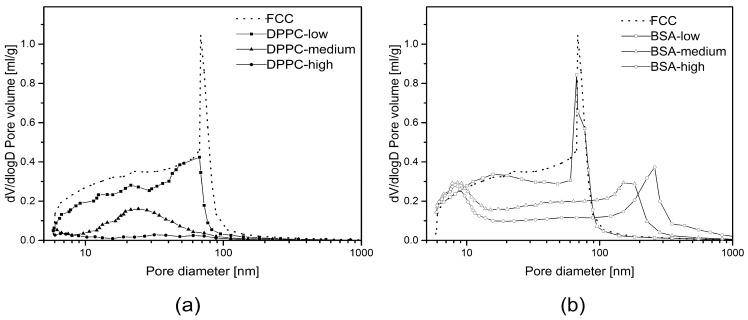
Pore size distribution obtained from mercury intrusion porosimetry. (**a**) Dipalmitoylphosphatidylcholine (DPPC) and (**b**) bovine serum albumin (BSA) loaded functionalized calcium carbonate (FCC) particles. Drug loads are defined in Table 1. Reference material was unloaded FCC. Samples were consolidated to exclude interparticulate pores. Smoothed curves represent means of *n* = 3 measurements.

To exclude interparticulate voids from the measurement, 200 mg of loaded FCC was consolidated to a 13 mm flat tablet applying 37 MPa using a manual hydraulic press (4350 L, Carver Ink., IN, USA). Pore size distribution of the samples was measured on a mercury intrusion porosimeter (Autopore IV, Micromeritics, Norcross, GA, USA). A 3 cm^3^ penetrometer with an intrusion volume of 0.387 mL and a stem volume of 0.412 mL was used. Pressures ranged from 3.59 to 206.64 kPa for low pressure intrusion and from 206.64 kPa to 206.78 MPa for the high pressure intrusion. The equilibration time was 10 s for both, the high and low pressure intrusion [29]. The pressure P that is required to push mercury into a capillary with a given diameter can be described by the Young–Laplace equation (Equation (10)) [34]:

(10) $$P=\frac{-4\times \gamma \times cos\theta }{d},$$

where γ is the surface tension (mN/m) of the penetrating liquid, θ is the contact angle (deg) of the liquid to the porous material, and d is the diameter [m] of the capillary. Figure 1b shows the principle behind mercury intrusion porosimetry. Using this experimental setup, pores ranging from 5 nm (min) up to 360 μm (max) could be measured. Additionally, information about the total pore volume or porosity, skeletal density, and the specific surface area of a sample was obtained (AutoPore Software v1.09, Micrometrics). All calculations were done based on raw data. For visual representation only (Figure 2), a moving average function with a period of 5 was applied to smoothen intrusion curves. The moving average function was not applied to regions showing extrema.

### 2.4. FIB-SEM

Samples were mounted on carbon adhesives, fixed with silver paste, and sputtered with 20 nm gold (EM ACE600, Leica, Wetzlar, Germany). The FIB-SEM (Helios NanoLab 650, FEI, OR, USA) consisted of an electron emission gun and a focused gallium ion beam column. The two sources were placed at an angle of 52° relative to each other. To protect the surface of the samples from damage by the ion beam, we first applied a 200 nm thick platinum band at the location of the cut. The stage was then tilted in the microscope to 52° so that it was perpendicular to the ion beam. Trenches with a final size of 20 µm width, 12 µm length, 10 µm depth were milled, using a 21 nA ion beam current to reveal cross sections of the sample. Milling time required for these settings was approx. 20 min. From each formulation, one batch was selected and three cross sections at least 20 µm apart were visualized. Cross sections were subsequently polished with a 100 pA ion beam. To reduce artifacts and charging effect on the images, the cross sections were sputtered with 3 nm platinum prior to imaging. Additionally, it is important to keep the samples fixed on the stubs during the milling process. The ion beam current had to be set to the lowest possible values for processing material to avoid sample ablation. Images were acquired from three different detectors: In Chamber Electron Detector (ICE), Everhart Thornley Detector (ETD) and Through Lens Detector (TLD). All images were taken by collecting secondary electrons (SE mode). Acquired images were corrected for tilt (−38°). Figure 1a shows a schematic representation of the FIB-SEM.

### 2.5. Image Analysis

For image analysis, FIJI distribution of ImageJ software was used [35]. From each cross section, the largest possible region of interest (ROI) without physical presence of foreign objects (e.g., external walls or ion depositions) was selected. A stripes filter with Daubechies wavelets (DB15) and a damping coefficient of 4.00 was used to remove curtaining effect [36]. To remove brightness gradients in the images, an FFT bandpass filter was applied. For segmentation of the images, a Python (v. 3.6.1, Continuum Analytics, Inc. Austin, TX, USA) code for SLIC based Superpixel Segmentation was used [37]. From these binary images, visually detected outliers were excluded, assuring that each cross section has at least one image included for calculations. The 2D porosity and average pore area were calculated using the particle analyzer function of ImageJ. To calculate pore volumes, pores were assumed to be spherical for the purpose of calculation. Therefore, absolute values of the pores cross sectional area that was obtained by image analysis, was used as area of a disc. The radius of this disc was then used to calculate the volume of a sphere. Bin sizes for pore size distribution were chosen in the same range as for MIP. The specific pore volume *V_SP_* [mL/kg] was calculated from image analysis using Equation (11):

(11) $$V_{SP}=\frac{V_{PI}}{W_{I}\times H_{I}\times D_{AV}\times \rho_{T}},$$

where *V_PI_* is the total pore volume (mL) that is created by the pores of the image, *W_I_* (m) is the width and *H_I_* (m) is the height of the image, and *D_AV_* (m) is the average pore diameter from the same image. The density of the tablet ρ*_T_* (kg/m^3^) was used to calculate the weight (g) of the sample.

### 2.6. Contact Angle Measurement

All measurements required to calculate the contact angle between the loading solutions and the surface of FCC were performed under atmospheric pressure at a temperature of 39 °C and 36 °C for DPPC-methanol and BSA-water, respectively. Densities of the loading solutions were measured using 3 mL glass pycnometers. Viscosity of the two solutions was measured with an Ubbelohde viscosimeter (Size 0, Paragon scientific Ltd., Wirral, UK).

Surface tension and sorption measurements were performed on a tensiometer (T100, Krüss, Hamburg, Germany). Surface tension was measured using the Wilhelmy plate method. For the sorption measurements, a sample holder with an inner diameter of 9 mm was filled with 900 mg of FCC and subsequently tapped until the powder bed was consolidated to a final height of 45 mm. N-hexane was used to determine the capillary constant. Liquid uptake was plotted as squared mass [g] against time [s] and the contact angle [deg] was calculated using Equation (12):

(12) $$cos\theta =\frac{m^{2}\times \eta }{c\times \rho^{2}\times \sigma \times t},$$

where *m* is the mass of the absorbed liquid (g), *t* is time (s), η is the viscosity (MPa·s), ρ is the density (g/mL), σ is the surface tension (mN/m) of the solutions, and *c* is the capillary constant of FCC. All measurements were done in triplicate.

### 2.7. Solubility

The solubility of BSA in water was determined by agitation of a saturated solution for 4 h at 36°C in a 1.5 mL Eppendorf tube, ensuring presence of undissolved BSA particles. Subsequently, the samples were centrifuged for 30 min at 14,000 rpm (5415 C, Eppendorf, Hamburg, Germany), the supernatant was diluted, and the concentration was measured in a spectrophotometer (Spectramax M2, Molecular Devices, San Jose, CA, USA) at 280 nm in a 10 mm cuvette. A calibration line with a linear regression of *R*^2^ > 0.999 was used to determine concentrations.

To determine the solubility of DPPC, the material was added in approximately 100 mg portions into glass vials containing 3 mL of methanol. The vials were kept at 39 °C in a water bath. Due to high viscosity, the experiment was stopped after adding 3.3 g of DPPC. Experiments were as well carried out at 50 °C, which is the temperature at the final stage of the evaporation process, when cooling due to evaporation becomes insignificant.

## 3. Results

### 3.1. Initial Loading Solution Properties

Table 1 summarizes physical properties of loading solutions at the start of the evaporation process. Solubility of DPPC in methanol was >1.1 g/mL at 39 °C. However, at 50 °C, only 300 μL of methanol was necessary to turn 1 g of solid DPPC into a clear, highly viscous mass. Pure DPPC does not melt at 50 °C. The solubility of BSA in water at 36 °C was 0.392 g/mL.

### 3.2. Loading

FCC was loaded by solvent evaporation with low, medium and high amounts of DPPC (using methanol as a solvent) and BSA (using water) as model substances. The content of the loaded substances in the samples was measured by thermogravimetry. The results were 5.2 ± 0.1% (Low), 21.3 ± 0.8% (Medium), and 29.5 ± 3.6% (High) (*w*/*w*) for BSA samples and 5.8 ± 0.2% (Low), 25.0 ± 0.9% (Medium), and 38.6 ± 0.8% (High) (*w*/*w*) for DPPC samples. Total yield was >90% (*w*/*w*) for all formulations. After milling and sieving, all formulations appeared as fine white powder. Results of TGA are available in supplementary data, Figure S1. 

### 3.3. Mercury Intrusion Porosimetry

Figure 2 shows MIP plots for BSA and DPPC loaded FCC. Both plots show a decrease in total pore volume at increasing loads. At the highest loading, the total pore volume is reduced to approximately 11% and 61% (*v*/*v*) of the pure FCC for DPPC and BSA, respectively. MIP showed different evolution of the pore size distribution while increasing the amount of loaded substances. While the pore volume below 10 nm is reduced significantly for the DPPC samples, BSA loaded FCC does not show any change at this range. At medium and high loadings, BSA samples show a shift of pore size distribution towards larger pores, indicating the formation of additional pores between 100 and 1000 nm. In contrast, the volume decreases for the entire population of pore sizes in DPPC samples but there is no sign for the formation of new pores. This is also visible when looking at the main peak between 60 and 100 nm, which disappears in case of DPPC but remains nearly unchanged for BSA at low loads. At the highest loading of DPPC, MIP shows almost no residual pore volume anymore.

### 3.4. FIB-SEM and Image Analysis

**Figure 3:**
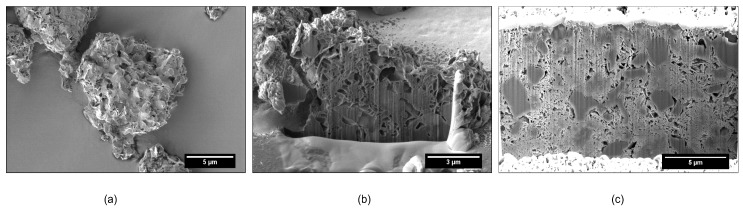
Visualization of unloaded FCC; (**a**) SEM analysis of an individual FCC particle; (**b**) focused ion beam scanning electron microscopy (FIB-SEM) based visualization of a cross section of an individual particle; (**c**) unloaded FCC after consolidation at 37 MPa.

**Figure 4:**
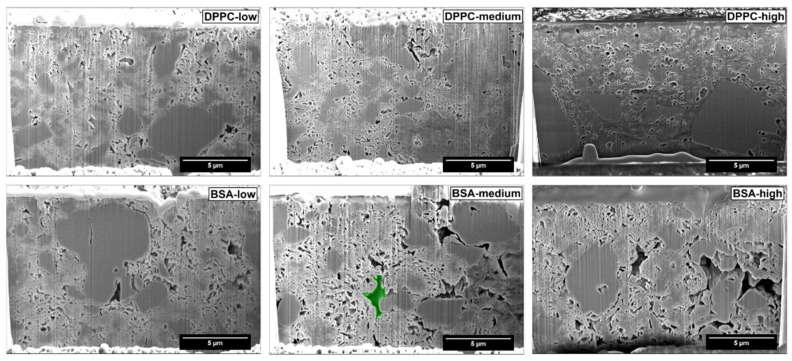
Analysis of consolidated FCC particles loaded with DPPC or BSA. Particles loaded with DPPC (**top row**) or BSA (**bottom row**) were analyzed by FIB-SEM. Mercury intrusion porosimetry results for the same material are shown in Figure 2. An example showing an interparticulate void is highlighted in green.

Figure 3 and Figure 4 show SEM and FIB-SEM images of unloaded FCC, or FCC loaded with BSA, or FCC loaded with DPPC. Apart from the porous structure, solid non-porous cores of calcium carbonate are visible in the images.

The SEM images showed reduction in pore size in DPPC formulations at higher loading. The pores were smaller, isolated, and had a different morphology than in unloaded FCC (Figure 3), with the FCC lamellae almost not distinguishable anymore. Figure 4 shows that the change in pore size or morphology is almost not detectable at lower loadings. Images show clearly the occurrence of interparticulate voids for FCC loaded with BSA, which are not present in case of DPPC.

**Figure 5:**
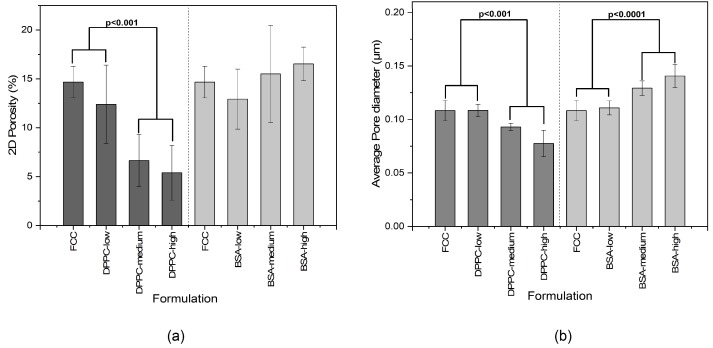
Quantitative assessment of porosity and pore diameter of FCC formulations. Results of (**a**) calculated 2D porosity and (**b**) average pore diameter from analysis of FIB-SEM images from different formulations of DPPC and BSA loaded into FCC. P-Values were calculated from one way ANOVA and Bonferroni post hoc test. Values are means ± SD, (*n* ≥ 3).

Figure 5 shows the quantitative assessment of results from FIB-SEM image analysis. Porosity of FCC was reduced with increasing loading of DPPC, while BSA loaded samples had no obvious trend of change in total porosity. The average pore diameter for both materials was almost unchanged at low loadings. At medium and high loadings, DPPC loaded FCC showed reduced pore size (*p* < 0.001) and BSA loaded FCC showed increase in pore diameter (*p* < 0.0001).

**Figure 6:**
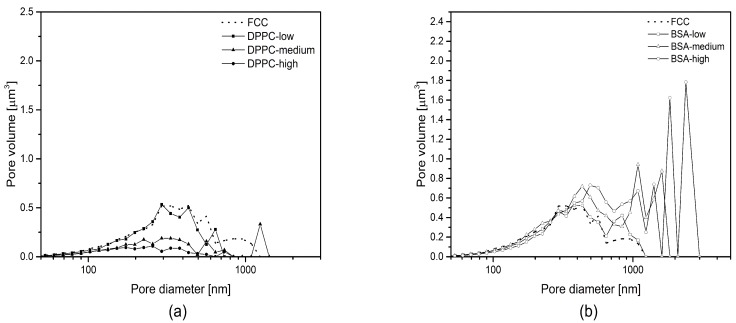
Pore size distribution based on FIB-SEM image analysis. Calculations were done assuming spherical pore shapes; (**a**) DPPC low, medium, and high loadings were compared to unloaded FCC; (**b**) BSA low, medium and high loadings were compared to unloaded FCC. Large voids (pore diameter > 1000 nm) in higher loadings of BSA are indicative of external crystallisation of BSA. Values are means of *n* ≥ 3.

Figure 6 shows calculated pore size distributions based on image analysis. The difference in pore diameter obtained from MIP (Figure 2) and SEM (Figure 6) is due to different pore shape assumptions in the two methods (cylindrical in MIP and spherical in image analysis). Average pore size (Figure 5) and pore size distribution from image analysis (Figure 6) were in accordance with MIP, showing larger sized pores at higher BSA loading. A trend of pore reduction in all sizes in the DPPC samples was observed. However, for the highest loading of DPPC, mercury intrusion results indicated complete pore filling, while the FIB-SEM images of this formulation showed presence of smaller, inaccessible pores.

**Figure 7:**
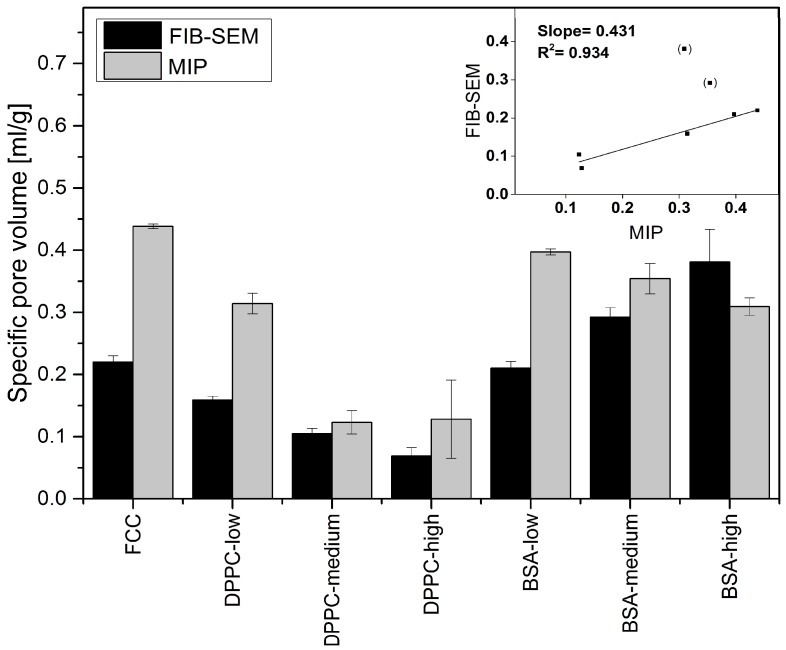
Specific pore volumes of FCC formulations. Pore volumes were calculated based on FIB-SEM image analysis (black) or by MIP (grey). Insert: correlation plot for both methods. Values are means ± SD, (*n* ≥ 3).

Figure 7 shows the specific pore volumes (mL/g) obtained by the two different methods. Data from MIP suggests, that the specific pore volumes decrease with increasing *DL* from 0.438 (unloaded FCC) to 0.128 and 0.309 mL/g for DPPC-high and BSA-high, respectively. Image analysis shows a reduction of the specific pore volume from 0.22 (unloaded FCC) to 0.069 mL/g for DPPC-high, but an increase up to 0.381 mL/g for BSA-high. The correlation coefficient of the two methods is *R*^2^ = 0.934 and the slope of the trend line is 0.431 (Figure 7, top right corner). The two data point in brackets (BSA-high and BSA-medium) are excluded from the linear fit due to observed external crystallization.

## 4. Discussion

Porous microparticles such as FCC can be used as carriers for drugs and other materials. In the present study, FCC particles were loaded by an established solvent evaporation method [17] using two model compounds: a hydrophilic macromolecule (BSA with a molecular weight of 66 kDa) and a small and lipophilic compound, i.e., DPPC. Loading efficiency was over 90% for both compounds and a drug load of 30% and 38% was measured for BSA and DPPC, respectively. Despite these similarities with respect to loading efficiency and loading capacity, the actual mechanism of drug loading is clearly different. The loading solution for DPPC shows a lower surface tension than the one for BSA as well as a lower viscosity. Due to the resulting lower contact angle, DPPC penetrates and solidifies within the pores. This notion is supported by the following observations: First, MIP as well as image analysis show gradual pore volume reduction. This applies to pores smaller than 500 nm. Second, DPPC does not lead to formation of additional interparticulate pores due to major penetration/deposition into the porous meshwork. In contrast, BSA is deposited solely on the surface of particles. This is supported by the observation that no filling of pores <500 nm can be detected by image analysis. In addition, upon loading, voids with a size of over 500 nm are created in between particles representing external crystallization. The occurrence of external crystallization can be explained by the solubility of the model substances. DPPC is highly soluble in methanol. In contrast, during the evaporation process, BSA reaches its solubility limit, when the volume of the remaining water is approx. 2.3 mL. At this point, only approx. 40% (*v*/*v*) of the BSA-solution is inside the pores of the FCC particles leading to external deposition of precipitated BSA.

Our results clearly indicate, that the methods used (i.e., FIB-SEM and MIP) have to be combined to give a realistic picture of the inner structure and porosity of the studied particles. FIB-SEM provides a detailed insight into the inner structure of FCC. The structure of the lamella and the solid starter cores is consistent with the process of FCC production explained by Levy et al. [38]. Pores of various sizes and shapes can be easily distinguished. Upon loading, filling of intraparticle pores and the formation of surface deposits can be documented.

Surprisingly, the obtained results did not necessarily correlate with results from mercury intrusion porosimetry. For example, average pore sizes obtained by both methods were very different. The average pore diameters of pure FCC determined by image analysis and MIP are 108 and 23 nm, respectively. This considerable difference can be explained by the ink bottle effect during MIP measurement. MIP assumes a cylindrical pore shape, while image analysis reveals the actual size of the pores, which in fact can consist of a small opening being connected to a much larger inner body. Thus, image analysis is giving information about the diameter of such pores bodies, while MIP is reporting the largest opening (throat) towards a pore. The ratio (4.7) between the average pore diameters obtained by the two different methods might therefore reflect the geometrical ratio of the body diameter of a pore to its throat. With respect to the specific pore volume, FIB-SEM and MIP show a good correlation (R^2 = 0.934). This does not apply to BSA formulations with a *DL* > 5% (*w*/*w*) due to the aforementioned external crystallization leading to the formation of interparticle pores. However, 2D imaging can only visualize a cross-section of a particle. The negative correlation plot (Figure 7) indicates that the pore volumes obtained by image analysis might be underestimated by a factor of 2. This can be attributed to the assumption of a spherical pore geometry when calculating pore volumes from 2D images. Selecting the right pore geometry is therefore crucial for reliable measurements based on image analysis. Based on images of the internal structure of FCC (Figure 3b) it seems obvious, that other pore geometries such as frustum, bipyramid or other polyhedra might describe the pore geometry much closer. It remains, however, to be determined if such a detailed structural analysis would be an added value.

MIP is a fast, precise, and accurate method for the analysis of materials that contain accessible and interconnected pores, but becomes less accurate when pores are isolated, e.g., due to clogging. MIP requires rather large sample sizes (0.1–1 g) and interpretation must respect the incapability of the machine to register isolated pores. MIP should therefore be combined with other methods such as flow imaging microscopy, thermoporometry, and teraherz time domain spectroscopy [30,39,40]. In contrast to these alternative methods, however, FIB-SEM is able to directly visualize the internal structure of a porous material with a resolution of several nanometers. Results are not influenced by pore accessibility or connectivity or type of loading [41]. Another advantage of FIB-SEM is its possibility to be coupled with an EDX detector, which allows to differentiate between structural components of a multiple component carrier system, deposits within pores and the surrounding structural material.

Compared to MIP, the range of materials that can be studied with FIB-SEM is wider. Considering the high pressures (up to 400 MPa) acting on the samples during a MIP analysis, one cannot exclude damages to the sample, e.g., deformation or fracture. However, image analysis has limitations since it is applicable only for a small area of the sample. For geometrically complex structures such as FCC, not all pores are reliably recognized by the algorithm. This requires extensive manual work for image processing. More repetitions are therefore needed to get statistically relevant data. Here, MIP has a clear advantage over image analysis, since the sample size (number of pores) is much larger (approx. factor 10^4). However, investigation of the geometrical properties of the porous structure, which is essential to study liquid flow in porous media especially in clogged pore conditions, is impossible without micro tomographic sectioning using FIB-SEM.

## 5. Conclusions

As both FIB-SEM image analysis and MIP each have their limitations, we propose that both methods should be combined to allow for accurate interpretation of substance distribution within porous microcarriers. Using such a combined approach, we could for the first time visualize the internal structure of FCC and clearly discriminate between open, blocked, and drug loaded pores.

## Figures and Tables

**Table 1:** Physical properties of loading solutions. For density, viscosity, and surface tension, values are means ± SD (*n* = 3). Contact angle was calculated based on values from the table and the averaged results from the sorption measurements (*n* = 3).

Loading Solution	Density (kg/m^3^)	Viscosity (MPa·s)	Surface Tension (mN/m)	Contact Angle (deg)
Methanol (DPPC-high)	789.0 ± 0.4	0.59 ± 0.01	22.0 ± 0.1	45.3
Methanol (DPPC-medium)	790.9 ± 1.9	0.57 ± 0.01	21.9 ± 0.1	24.2
Methanol (DPPC-low)	785.6 ± 0.2	0.54 ± 0.01	21.8 ± 0.1	34.9
Water (BSA-high)	1001.7 ± 1.1	0.82 ± 0.01	47.5 ± 0.3	53.4
Water (BSA-medium)	1002.0 ± 0.9	0.75 ± 0.01	48.7 ± 0.4	39.8
Water (BSA-low)	999.1 ± 0.1	0.69 ± 0.01	50.8 ± 0.5	66.3

## Supplementary Materials

The following are available online at http://www.mdpi.com/1999-4923/11/1/32/s1, Figure S1: Thermogravimetric results of pure FCC, BSA, DPPC, and the final formulations.
