# Supplementary material for: Loading of Porous Functionalized Calcium Carbonate Microparticles: Distribution Analysis with Focused Ion Beam Electron Microscopy and Mercury Porosimetry
Source: Pharmaceutics. 2019 Jan 15;11(1):32. doi: 10.3390/pharmaceutics11010032 (PMC6358859; doi:10.3390/pharmaceutics11010032)
Supplement: Supplementary file 1 [file pharmaceutics-11-00032-s001.pdf]

pharmaceutics

MDPI

Article

Loading of Porous Functionalized Calcium Carbonate Microparticles: Distribution Analysis with Focused Ion Beam Electron Microscopy and Mercury Porosimetry

Maryam Farzan [1,†], Roger Roth [1,†], Gabriela Québatte [1], Joachim Schoelkopf [2], Jörg Huwyler [1] and Maxim Puchkov [1,*]

[1] Division of Pharmaceutical Technology, Department of Pharmaceutical Sciences, University of Basel, Klingelbergstrasse 50, 4056 Basel, Switzerland; maryam.farzan@unibas.ch (M.F.); roger.roth@unibas.ch (R.R.); gabriela.quebatte@unibas.ch (G.Q.); joerg.huwyler@unibas.ch (J.H.)

[2] Fundamental research, Omya International AG, 4665 Oftringen, Switzerland; joachim.schoelkopf@omya.com

* Correspondence: maxim.puchkov@unibas.ch; Tel.: +41-61-207-1619

† These authors contributed equally to this work.

Received: 30 October 2018; Accepted: 10 January 2019; Published: 15 January 2019

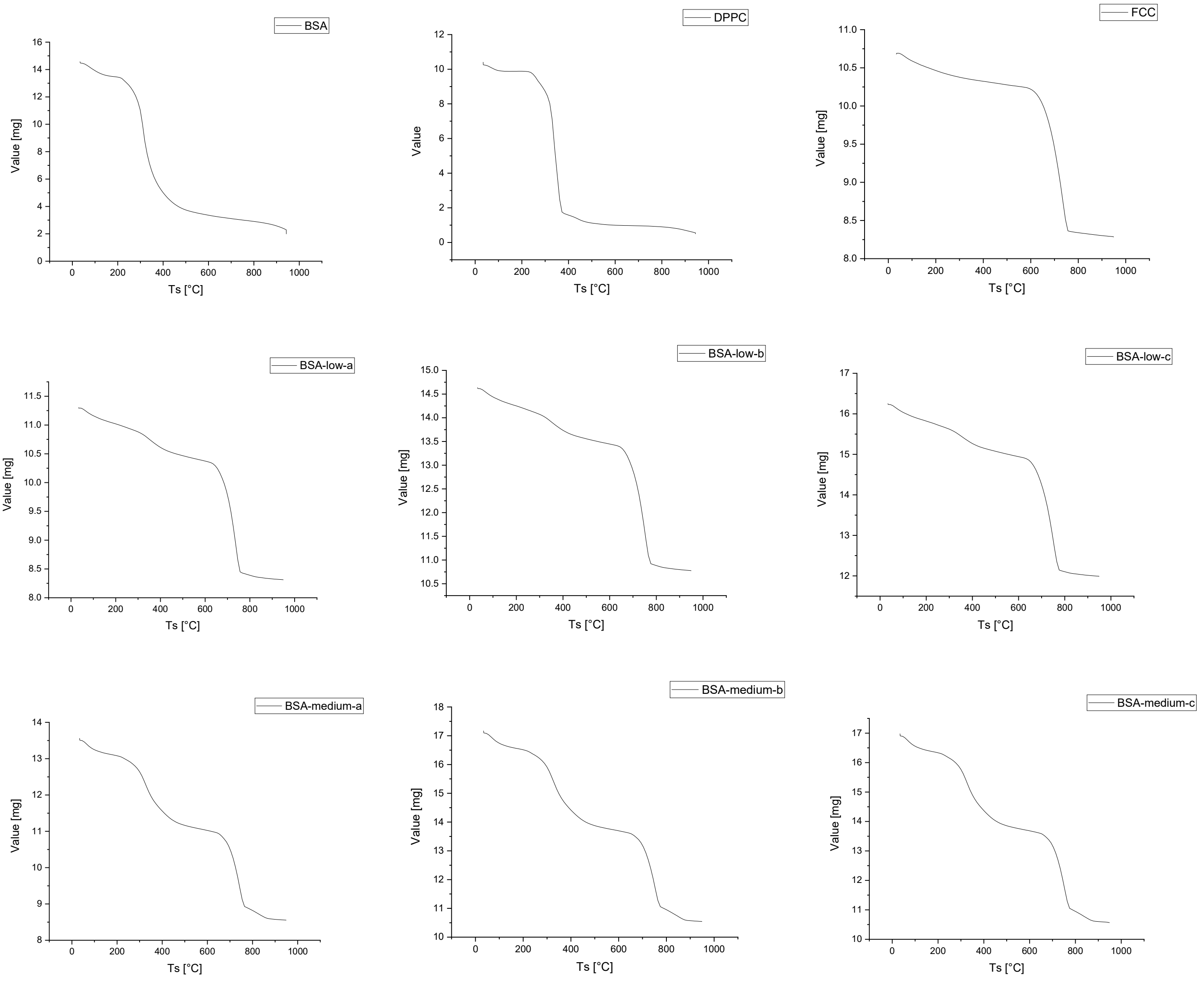

pharmaceutics

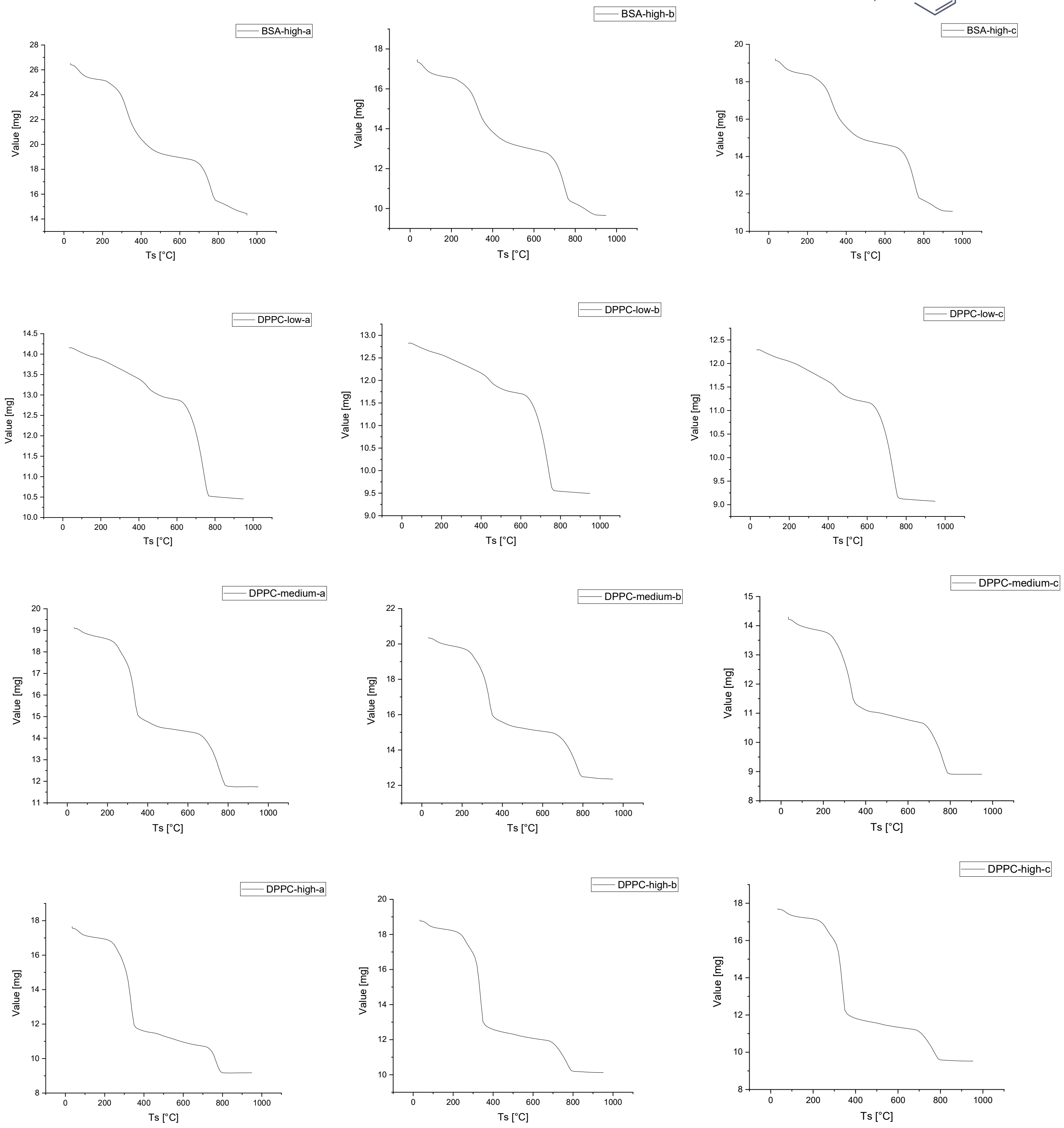

Figure 1-s: Thermogravimetric results of pure FCC, DPPC, and the final formulations. The results were obtained from three separate batches (a, b and c) of each formulation.
